# 3D modeling of in vivo MRI-guided nano-photothermal therapy mediated by magneto-plasmonic nanohybrids

**DOI:** 10.1186/s12938-023-01131-w

**Published:** 2023-08-01

**Authors:** Zahed Tavangari, Mohammadreza Asadi, Rasoul Irajirad, Abolfazl Sarikhani, Zahra Alamzadeh, Habib Ghaznavi, Samideh Khoei

**Affiliations:** 1 Finetech in Medicine Research Center, Iran University of Medical Sciences, Tehran, Iran; 2 Medical Physics Department, Iran University of Medical Sciences, Tehran, Iran; 3 Pharmacology Research Center, Zahedan University of Medical Sciences, Zahedan, Iran

**Keywords:** Image-guided therapy, MRI, Magneto-plasmonic nanohybrids, Numerical modeling, Nano-photothermal therapy

## Abstract

**Background:**

Nano-photothermal therapy (NPTT) has gained wide attention in cancer treatment due to its high efficiency and selective treatment strategy. The biggest challenges in the clinical application are the lack of (i) a reliable platform for mapping the thermal dose and (ii) efficient photothermal agents (PTAs). This study developed a 3D treatment planning for NPTT to reduce the uncertainty of treatment procedures, based on our synthesized nanohybrid.

**Methods:**

This study aimed to develop a three-dimensional finite element method (FEM) model for in vivo NPTT in mice using magneto-plasmonic nanohybrids, which are complex assemblies of superparamagnetic iron oxide nanoparticles and gold nanorods. The model was based on Pennes' bio-heat equation and utilized a geometrically correct mice whole-body. CT26 colon tumor-bearing BALB/c mice were injected with nanohybrids and imaged using MRI (3 Tesla) before and after injection. MR images were segmented, and STereoLithography (STL) files of mice bodies and nanohybrid distribution in the tumor were established to create a realistic geometry for the model. The accuracy of the temperature predictions was validated by using an infrared (IR) camera.

**Results:**

The photothermal conversion efficiency of the nanohybrids was experimentally determined to be approximately 30%. The intratumoral (IT) injection group showed the highest temperature increase, with a maximum of 17 °C observed at the hottest point on the surface of the tumor-bearing mice for 300 s of laser exposure at a power density of 1.4 W/cm^2^. Furthermore, the highest level of tissue damage, with a maximum value of Ω = 0.4, was observed in the IT injection group, as determined through a simulation study.

**Conclusions:**

Our synthesized nanohybrid shows potential as an effective agent for MRI-guided NPTT. The developed model accurately predicted temperature distributions and tissue damage in the tumor. However, the current temperature validation method, which relies on limited 2D measurements, may be too lenient. Further refinement is necessary to improve validation. Nevertheless, the presented FEM model holds great promise for clinical NPTT treatment planning.

**Supplementary Information:**

The online version contains supplementary material available at 10.1186/s12938-023-01131-w.

## Background

According to the statistics, cancer is the second leading cause of morbidity and mortality across the globe, which about half of the incident cases lead to death [1]. Given the high mortality rate of cancer, researchers have been pursuing more precise diagnostic modalities and effective treatment techniques [2]. Nano-photothermal therapy (NPTT) is a fast-growing technology that holds new promise for cancer treatment. NPTT takes advantage of the photothermal effect of photothermal agents (PTAs) that can absorb light and convert the absorbed electromagnetic waves into heat, leading to local overheating. The main advantages of NPTT compared to traditional cancer treatments include minimal invasiveness, minimizing damage to the surrounding normal tissues, and the potential of bioconjugation with various vectors to achieve more accurately and specifically targeted therapy [3, 4].

Recently, noble metal nanoparticles are considered as one of the potentially valuable PTAs because of their special characteristics such as localized surface plasmon resonance (LSPR), which converts light into heat through a cohort oscillation of conduction electrons induced by electric field of light. Gold nanorods (AuNRs) attracted attention the most among noble metal nanoparticles and widely studied due to their unique biocompatibility and optical properties [5–7]. In this work, AuNRs are used because of their tunable light absorbance peak, which can be adjusted by aspect ratio to resonate with near-infrared (NIR) light. The first NIR window (650–950 nm) is ideal for NPTT because it has more penetration depth and travels further in tissue, although due to strong scattering and lower absorption of laser interaction with tissue, the temperature rise is not enough for therapeutic purposes [8]. Plasmonic nanoparticles guarantee efficient temperature delivery, mitigating concerns regarding skin damage and overcoming the limitation of temperature rise [9–11].

Controlling temperature in NPTT is essential to avoid surface overheating and thermal damage to healthy tissues. Several methods have been proposed to adjust the temperature distribution in photothermal therapy to mitigate the unwanted variations in temperature, active control methods such as periodic heating strategy [12, 13] and minimum invasive method [14] adjust the thermal dosage by modifying the external heat source based on measured or predicted temperature during the treatment. A passive control method was introduced based on phase change nanoparticles, which maintain a uniform temperature in the targeted area. In spite of promising results in the passive method, in vivo studies were insufficient and needed further investigation [15]. The results of these studies concerning photothermal temperature distribution are incomplete, and further advancements are needed to enhance the accuracy of the model. The accuracy of in vivo NPTT simulation highly depends on the plasmonic nanoparticle distributions over the tumor area, the theory for calculating the extinction coefficient of plasmonic nanoparticles, and the mathematical model of light diffusion. None of the aforementioned studies considered the real spatial distribution of photothermal agents within the tumor. As far as we know, spatial temperature distribution based on our approach has not been reported to date.

**Fig. 1:**
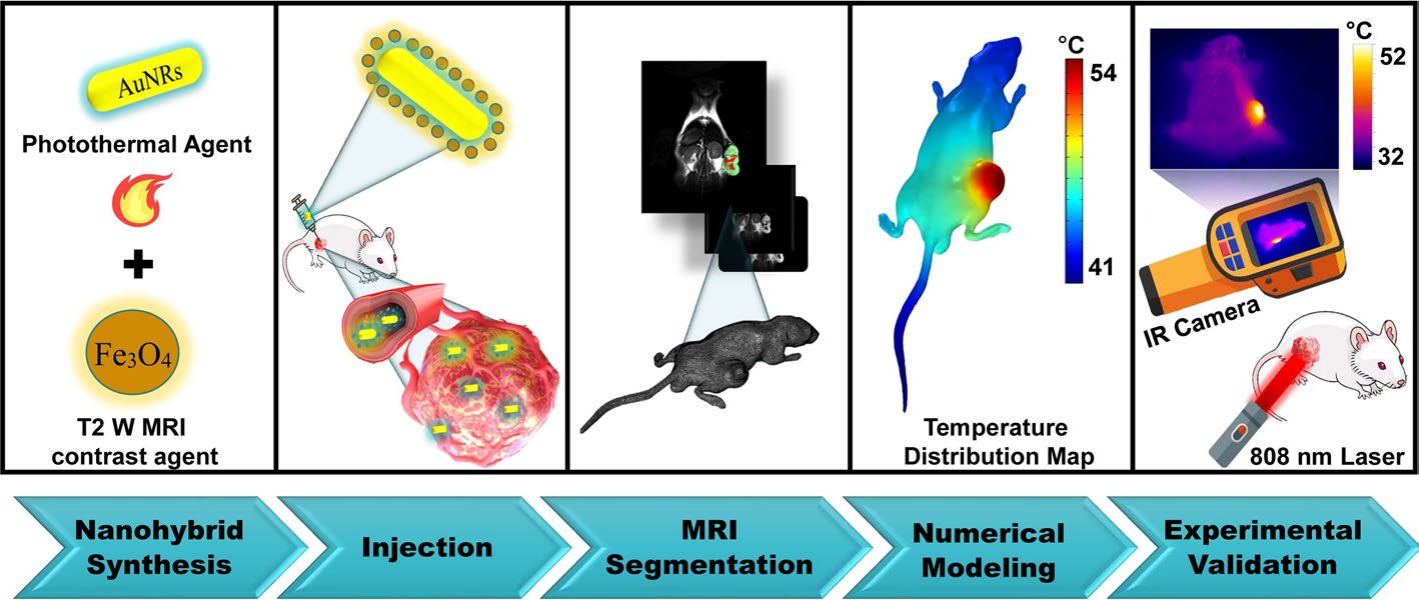
Schematic illustration of different steps of the present study

Herein, we present a 3D numerical modeling strategy for NPTT mediated by a nanohybrid made of a complex assembly of carboxymethyl dextran (CMD)-coated iron oxide NPs on bovine serum albumin (BSA)-coated AuNRs. On the one hand, iron oxide NP was included in this nanohybrid as it is a T2-weighted MRI contrast agent, and it can label the AuNRs to be observable in MRI images. In this way, MRI images make it possible to get insight into the distribution of nanohybrids over the tumor area, and they can be used as real data to build the geometry of 3D numerical model. On the other hand, we established a 3D in vivo model based on physical parameters of synthesized nanohybrids, using radiative transport theory, Pennes' bio-heat equation, and the Arrhenius equation. We believe that this model should be able to yield more accurate prediction of temperature distribution which will be useful for determining the dosage of nanohybrids, laser power, and exposure time that are required to predict irreversible tumor damage. Figure 1 presents a graphical overview of the current study.

## Results

### Characterization

**Fig. 2:**
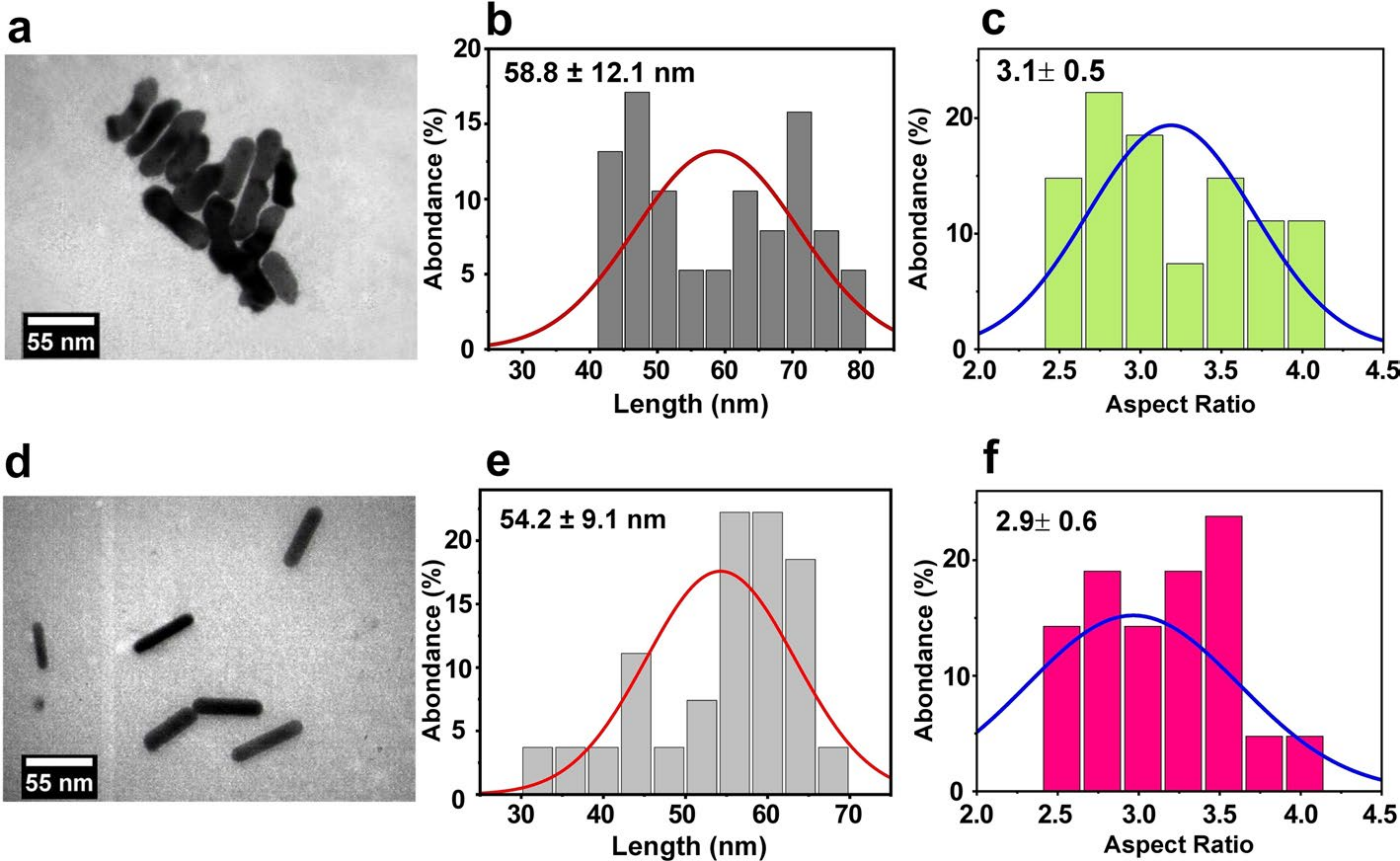
TEM images of (**a**) nanohybrids and (**d**) AuNRs. Length distribution of (**b**) nanohybrids and (**e**) AuNRs. Aspect ratio distribution of (**c**) nanohybrids and (**f**) AuNRs

Figure 2 indicates the morphological characterization of nanohybrids and AuNRs using TEM. Based on TEM measurements, the length of AuNRs was 54.2 ± 9.1 nm, and the length of nanohybrids was 58.8 ± 12.1 nm. Also, the aspect ratio of nanohybrids and AuNRs was 2.9 ± 0.6 and 3.1 ± 0.5, respectively. The irregular rod-shaped morphology in the TEM image of the nanohybrids, as shown in Fig. 2a, compared to the AuNRs as displayed in Fig. 2d, suggests a successful assembly of Fe_3_O_4_@CMD with AuNRs@BSA. This conclusion is supported by a battery of tests, including UV–visible, FTIR, and XRD, which all confirm the attachment between Fe_3_O_4_ and AuNRs.

**Fig. 3:**
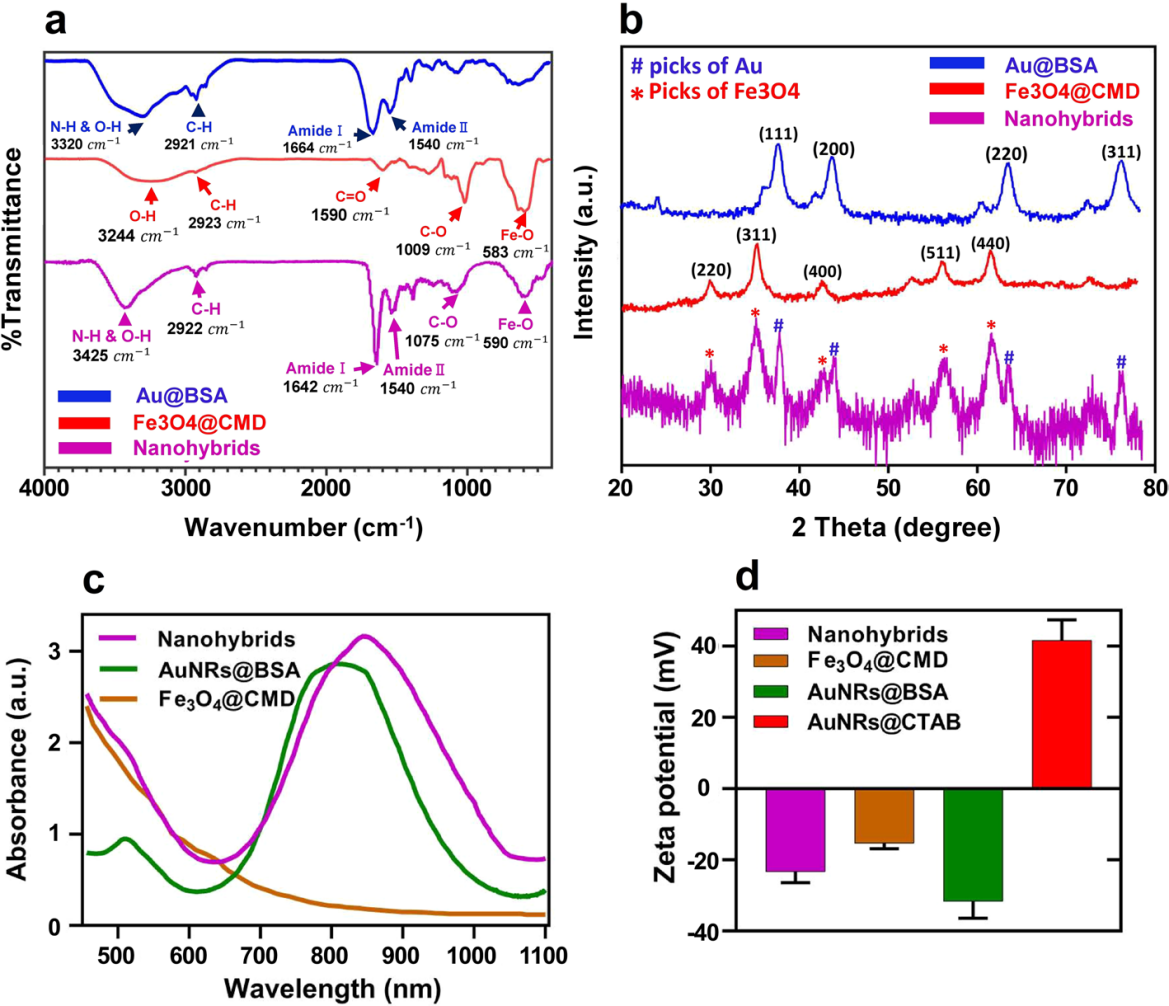
**a** FTIR spectra, **b** XRD diffractograms**, c** UV–visible absorbance spectra, and **d** zeta potential of prepared samples

Figure 3a shows the FTIR spectra of prepared samples. In Fe_3_O_4_@CMD spectrum, we observed the signal corresponding to the O–H bonds related to the water adsorber groups that appeared in 3244 cm^−1^. We also found two peaks of CMD at 2923 cm^−1^ and 1009 cm^−1^, which belong to C–H and C–O bonds, respectively. At 1590 cm^−1^, the stretching of the carbonyl group of the asymmetric carboxyl group was found, and the Fe–O signal appeared at 583 cm^−1^.

Furthermore, in the spectrum of AuNRs@BSA, we observed typical peaks of BSA near 1664 cm^−1^ and 1540 cm^−1^ attributed to amide I and amide II, respectively. In 1664 cm^−1^, the N–H and OH bands were found, and C–H stretches were observed at 2921 cm^−1^. The loss of S–H stretching in free BSA at 2360 cm^−1^ confirmed the adsorption of BSA onto AuNRs via gold and sulfur bonding. Finally, comparing the spectrum of the nanohybrids with AuNRs@BSA, the following bands/changes were observed. The N–H and O–H stretching vibrations of the nanohybrids shifted from 3320 cm^−1^ to 3425 cm^−1^ compared to AuNRs@BSA. At 1664 cm^−1^, the amide I band also shifted to a new strong band at 1642 cm^−1^. Moreover, the peak observed around 590 cm^−1^ indicated the presence of Fe–O linkage in the product. These results verified the conjugation between Fe_3_O_4_@CMD and AuNRs@BSA.

The XRD confirmed the presence of Fe_3_O_4_ (JCPDS card number 75-0033) and Au (JCPDS card number 04-0784) in the nanohybrids (Fig. 3b). The formation of AuNRs was confirmed using UV–visible spectroscopy, which showed two notable plasmon peaks, one at 510 nm and the other at 800 nm. The wide band of absorption was observed at approximately 450–600 nm for Fe_3_O_4_@CMD NPs. The red shift in the plasmon band observed at 840 nm after conjugation of Fe_3_O_4_ with AuNRs strongly suggests that this shift is due to the attachment of Fe_3_O_4_ nanoparticles to the AuNRs, as shown in Fig. 3c. The zeta potential measurements of AuNRs@BSA, AuNRs@cetyltrimethylammonium bromide (CTAB), Fe_3_O_4_@CMD, and nanohybrids were − 31.3 ± 4.7 mV, 41.3 ± 5.6 mV, − 15.3 ± 1.5 mV, and − 23.3 ± 3.0 mV, respectively (Fig. 3d).

### In vivo MRI

**Fig. 4:**
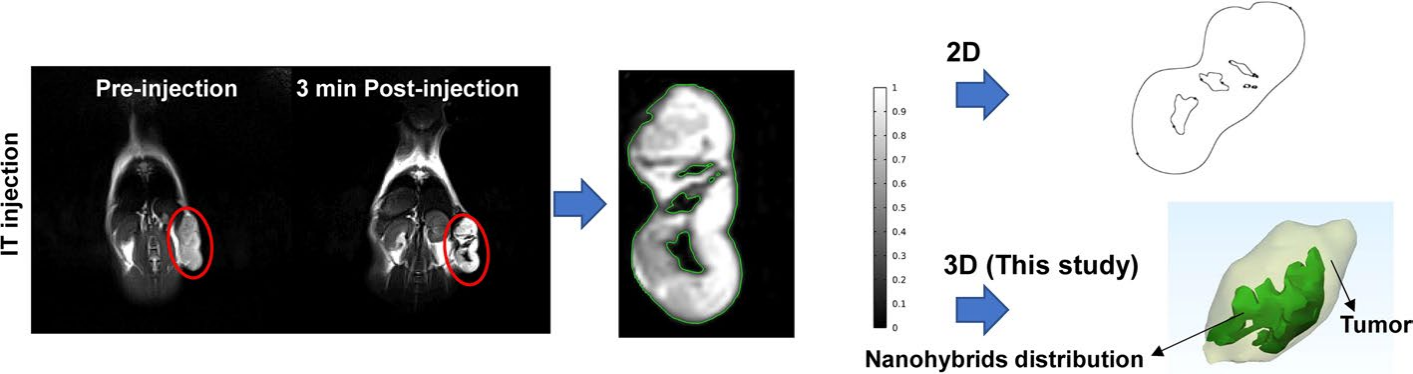
MR images of tumor-bearing mice (red contours indicate the tumor), and introducing possible approaches to determine the distribution of nanohybrids

We assessed the in vivo contrast-enhancing effect of nanohybrids in tumor-bearing mice. Figure 4 illustrates T2-weighted MR images obtained before and after injection. Following a 3-min post-injection interval, hypointense areas were observed in the intratumoral IT injection images. Furthermore, we performed a comparative analysis between our 3D approach and conventional 2D methods. These findings support the potential utilization of the synthesized nanohybrids in this study for MRI-guided NPTT.

### Gold nanoparticles biodistribution

The gold levels of nanohybrids, administered via intraperitoneal (IP) injection, within the dissected tumors were measured to be 1.9 ± 0.7 μg Au/g tissue, using inductively coupled plasma mass spectrometry (ICP-MS) analysis. This passive accumulation at the tumor site was observed 24 h post-injection.

### Temperature and thermal damage distribution

**Fig. 5:**
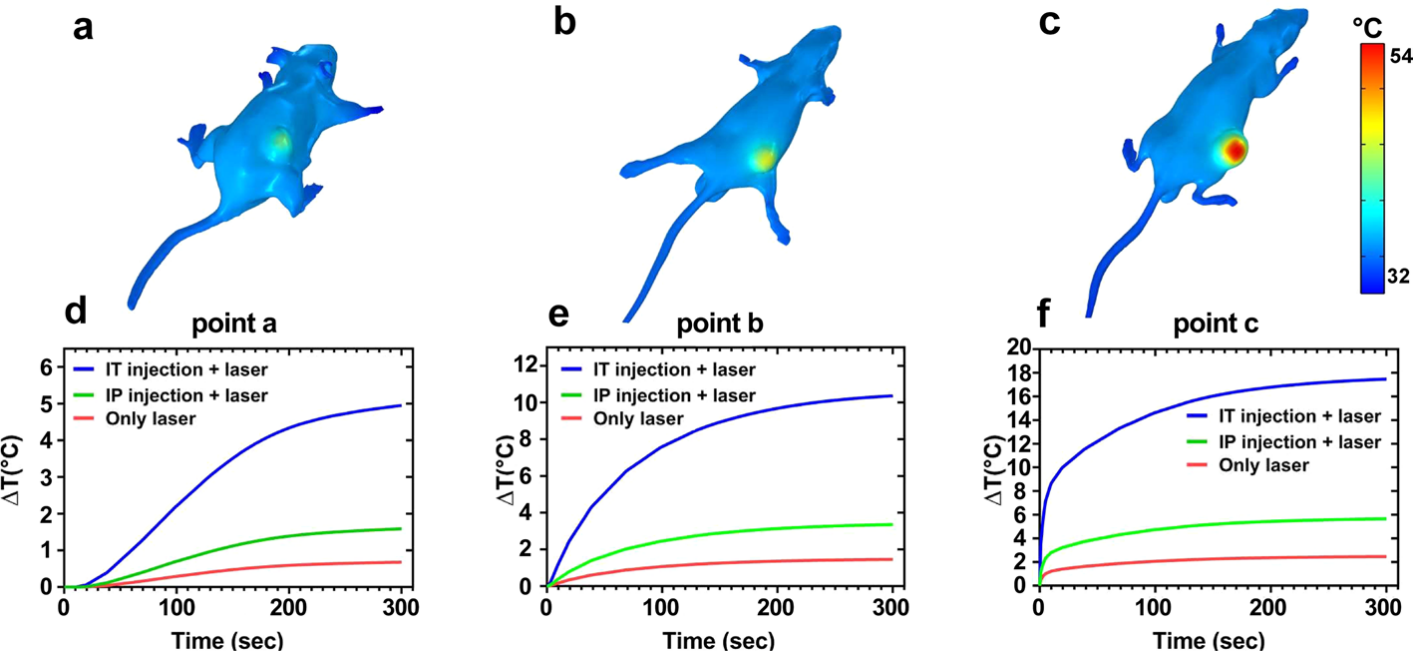
**a–c** Temperature distribution of three treatment groups: **a** laser alone, **b** IP injections + laser, and **c** IT injections + laser. **d–f** Temperature rise profile in different groups of mice at three different points after 5-min NIR laser irradiation (808 nm, 1.4 W/cm^2^)

**Fig. 6:**
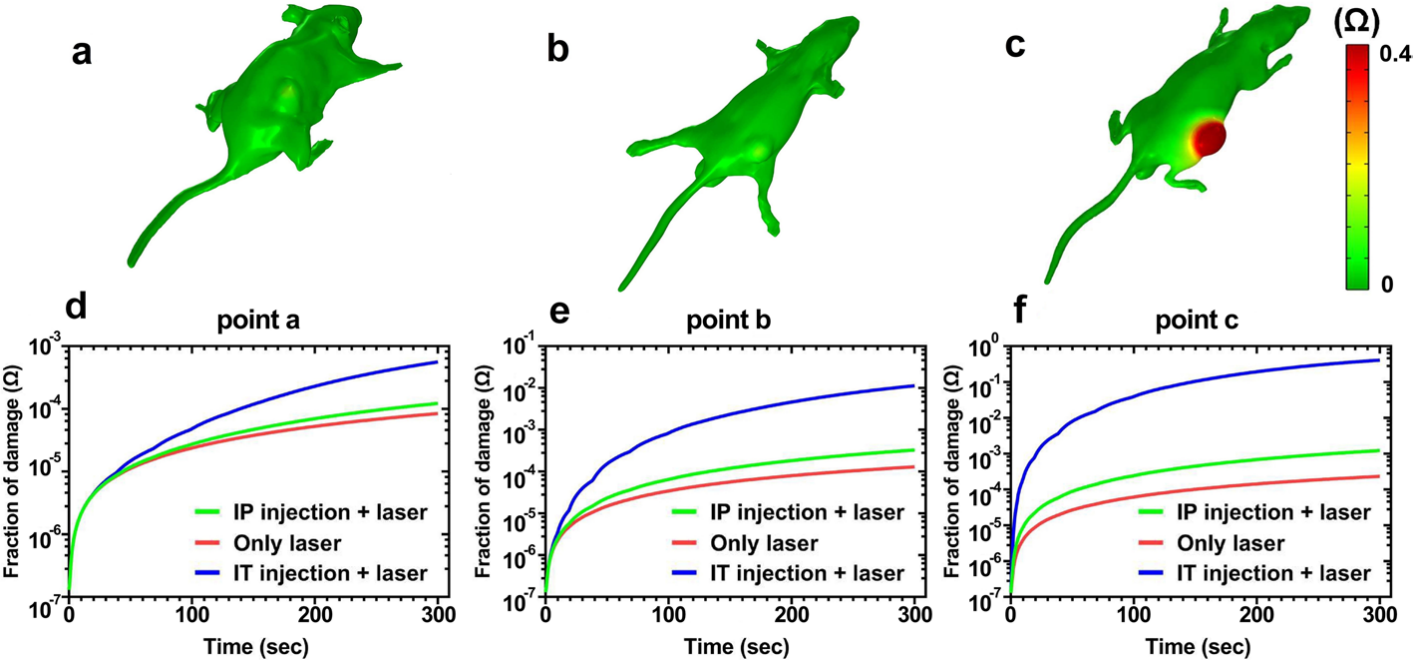
Thermal damage distribution of three treatment groups: **a** laser alone, **b** IP injections + laser, and **c** IT injections + laser. **d-f** Thermal damage rise profile in different groups of mice at three different points after 5-min NIR laser irradiation (808 nm, 1.4 W/cm^2^)

Based on the realistic geometry of the tumor-bearing mice and the distribution of nanohybrids within the tumor, 3D maps of temperature distribution (Fig. 5a–c) and thermal damages (Fig. 6a–c) were predicted. Furthermore, considering the defined points in Fig. 10, the temperature rise was calculated for three treatment groups at each point (Figs. 5d–f, and in 6d–f), thermal damages were predicted on the logarithmic scale for three treatment groups at each point. Results show that the tissue damage integral increases as the time of exposure is increased.

### Experimental validation of simulation results

**Fig. 7:**
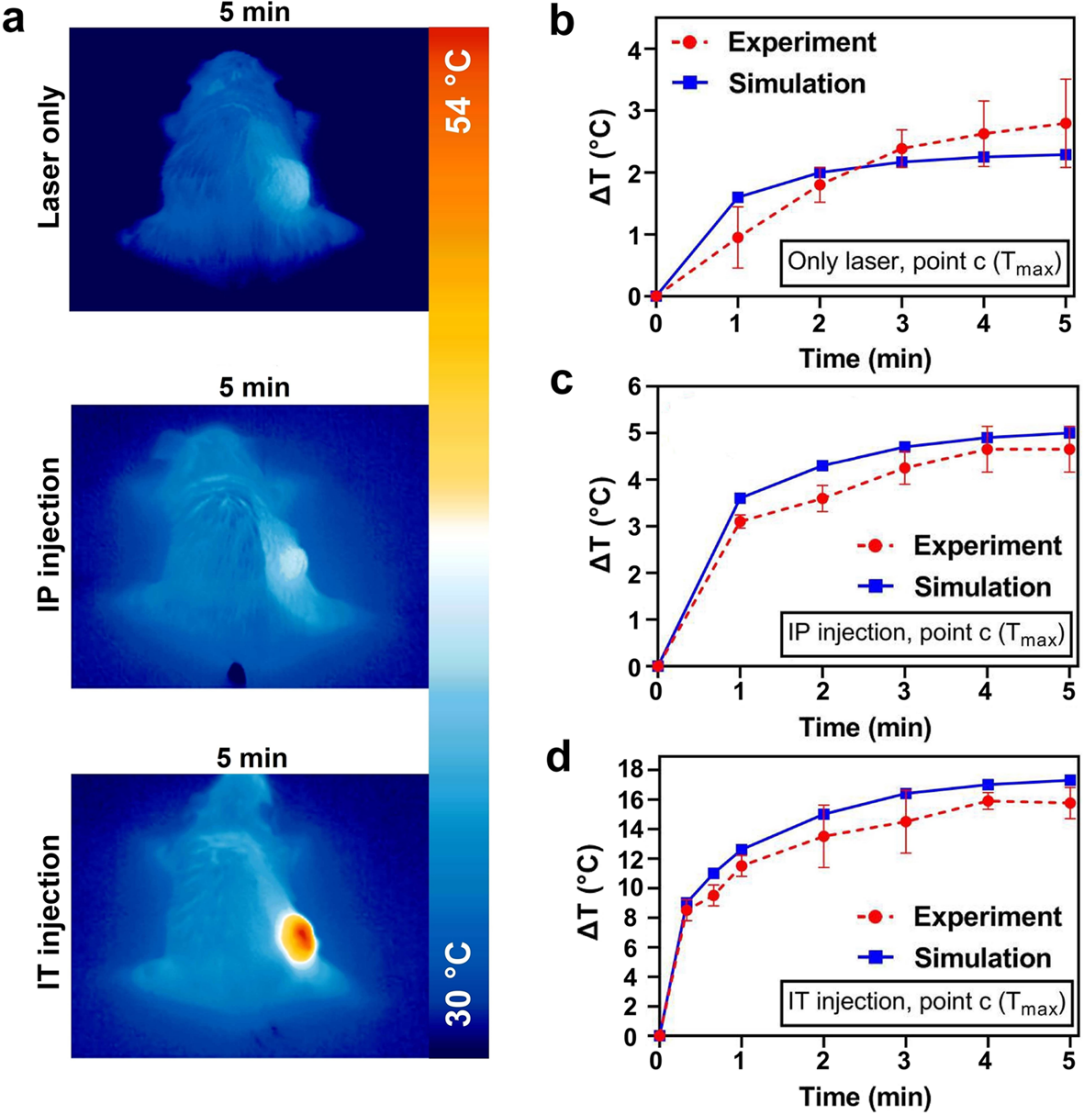
** a** Representative thermal images of tumor-bearing mice. **b-d** Comparison of temperature rise under 5-min laser irradiation (808 nm, 1.4 W/cm^2^) between simulation and experiment in three groups: **b** laser alone, **c** IP injection + laser, and **d** IT injection + laser, respectively. Error bars illustrate the standard deviation for 5 mice in each group

Among the three selected points in the tumor-bearing mice where simulations and computations were conducted, point c was chosen to assess the validity of the FEM numerical simulation results. Temperature measurements were performed using an infrared (IR) thermal imaging camera (Testo 875-1i, Germany) during 5 min of laser irradiation (808 nm, 1.4 W/cm^2^). Figure 7a illustrates IR camera images of mice during laser irradiation. Figure 7b–d shows the comparison of experimental and simulated temperatures. As shown in Fig. 7c–d, the simulations exhibited an overestimation of temperature rises in the injection groups. In the no injection group simulation (Fig. 7b), the temperature rise was either overestimated or underestimated. Nevertheless, the validation of the model demonstrated an acceptable level of accuracy in predicting temperature distributions. Error bars have been included for each experimental data point.

### In vivo NPTT

**Fig. 8:**
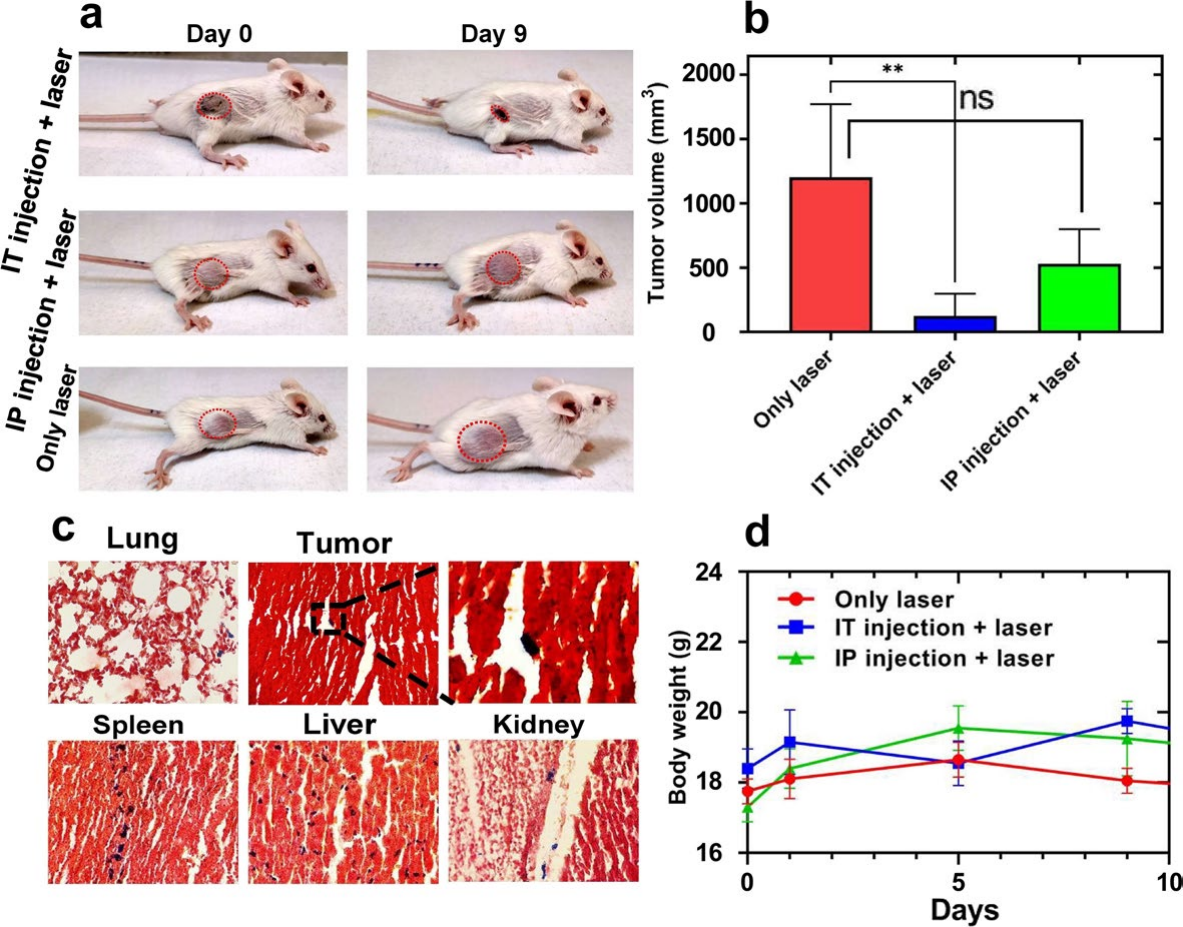
In vivo experiments. **a** Representative pre- and post-treatment photos of mice. **b** Tumor volume 9 days after treatment in various groups. **c** Prussian blue staining results were obtained for tumor, lung, spleen, liver, and kidney sections obtained from mice receiving IP injection. **d** Animal body weight changes in different groups of mice. (**p value < 0.001)

The data in Fig. 8b illustrate no statistically significant difference happened in tumor size between laser alone and IP injection + laser groups 9 days after treatment (Fig. 8a). While the tumor size in the IT injection + laser group significantly decreased compared to what was obtained for laser alone (*p* value < 0.001). Prussian blue staining results for the IP injection group showed a low amount of iron nanoparticles in the tumor, which was consistent with our observations in MRI study. We found no significant difference in body weight after injection of nanohybrids (Fig. 8d). However, additional investigations are necessary to evaluate biodistribution and potential toxicity.

## Discussion

We have developed a whole-body three-dimensional finite element model of in vivo NPTT mediated by magneto-plasmonic nanohybrids as an MRI contrast agent based on real geometry. The body mesh was constructed from a male BALB/c mouse as a representative for each treatment group, which maintaining the geometrical shape of the mouse body and nanohybrid distributions within the tumor. The model predicts temperature distribution and tissue damage in three dimensions across the mice body at high resolution. The validation of model shows acceptable accuracy in the prediction of temperature distributions. Furthermore, we have introduced a nanohybrid with both NIR-absorbing and MRI contrast agent characteristics, making them desirable for MRI-guided NPTT.

Although many numerical modeling studies have been carried out in the field of NPTT, the models were mainly validated through phantom studies and accomplished on the basis of using a simplified geometry which caused inconsistency between simulation results and experiment results [16, 17]. Few 2D computational simulation studies based on the models established by realistic geometry obtained from one slice of CT-scan [18, 19] or MRI images [20, 21] which were done only for one slice of MRI/CT images and other distribution of nanoparticles in the tumor sections were missing. The novel feature of this model is the realistic spatial distribution of nanohybrids, which takes into account the geometry and inhomogeneity of the accumulated nanohybrids in the tumor. In this study, we created a method to build a 3D model of the whole animal body to overcome previous limitations. As shown in Fig. 4, we can observe nanohybrids spatial distribution at every point inside the tumor compared with previous two-dimensional studies. To the best of our knowledge, this study is the first 3D modeling of NPTT based on realistic geometry and distribution of PTAs.

AuNRs were used as a NIR-absorbing agent not only due to their unique localized surface plasmon resonance, but also because of their functionalization with diagnostic and therapeutic agents through covalent and ionic binding [22], the cytotoxicity associated with the CTAB in the synthesis procedure of AuNRs is one of the main limitations of using AuNRs@CTAB in biomedical applications [23]. The cost-effective biological macromolecule used to coat the surface of AuNRs to enhance the biocompatibility, thanks to the effective thiol binding of BSA, which can be assembled to the gold surface, moreover terminated amine groups of BSA remained untouched to react with carboxylic groups [24]. The synthesized AuNRs@BSA can fulfill our expectations in terms of photothermal point of view. However, we need an extra component for tracking injected PTAs. Let us discuss the magnetic properties of these nanohybrids, noninvasiveness and high spatial resolution of MRI motivated us to use this powerful imaging modality [25]. We synthesized Fe_3_O_4_ due to their relatively low cost and biocompatibility [26] coated with CMD as an MRI contrast agent. In order to attach AuNRs with Fe_3_O_4_, we conjugated carboxy groups of CMD with amine groups of BSA. Although the amine group of AuNRs@BSA has an electrostatic attraction to the carboxyl group of Fe_3_O_4_@CMD, the covalent amide bond formation was achieved by bioconjugation reactions [27]. In terms of safety considerations of the nanohybrid, we have addressed the issue of temperature rise and the potential risk of thermal damage in our results. However, for successful clinical translation, a comprehensive safety evaluation encompassing tests such as biocompatibility, toxicity, and long-term effects is essential.

Quantitative comparisons of PTAs pose challenges due to complexities arising from variations in material properties and experiment setups. Previous literature reports have indicated a photothermal conversion efficiency (PCE) of approximately 21% for AuNRs [28, 29]. Through the bioconjugation of AuNRs with Fe_3_O_4_, we have successfully increased this value to around 30%. Our nanohybrid demonstrates a comparable PCE to specific photothermal agents, including Au/polypyrrole@Fe_3_O_4_ nanocomposites (24%) [30], HCuS@Cu2S@Au (35%) [31], and BiVO_4_/Fe_3_O_4_@polydopamine (33%) [32]. It is important to note that a higher PCE value alone does not necessarily determine the ideal photothermal agent. Some studies have reported higher PCE values (more than 75%) by nanoparticles of larger dimensions [33, 34] in comparison to our work, which may pose limitations on their suitability for in vivo applications [29]. Furthermore, the lack of information regarding photothermal heating/cooling cycles in certain studies hampers the assessment of their stability [33, 34].

The present model has some limitations; first, the precise quantity of nanohybrid distributions (gold concentration map) could have been investigated noninvasively by magnetic particle imaging (MPI) due to its magnetite content because the ratio of AuNRs to Fe_3_O_4_ is a known parameter we can estimate the gold content from Fe_3_O_4_ concentration [35]. However, interesting approaches have been introduced for quantifying gold concentrations map based on CT-scan [36, 37], MPI approach performs nonionizing radiation, and it is a much more sensitive modality [38]. Furthermore, one of the objectives of this study was to validate the capability of synthesized nanohybrids in NPTT, not to introduce a reliable method for quantifying the gold content accumulated in tumor noninvasively. To this end, we quantified the gold contents via ICP-MS analysis, and the treatment simulations were validated based on these quantities; second, the accuracy of thermal simulations within tumor-bearing mice could have been improved through the validation of precise 3D temperature distribution using magnetic resonance (MR) thermometry. Unfortunately, the absence of a dependable validation tool limited validation to surface temperature only (2D). Nonetheless, the incorporation of 3D temperature mapping in future simulations has the potential to enhance temperature distribution predictions, promoting more effective treatments for NPTT.

## Conclusion

A three-dimensional finite element model of in vivo nano-photothermal therapy with a geometrically correct mice whole-body was developed. This model predicts tissue damage and temperature distributions across the mice body at high resolution and takes into account the geometry and inhomogeneity of the accumulated nanohybrids in the tumor based on MRI. Predicted points (A, B, and C) temperatures were compared with measured values. The comparison indicates that the model prediction is accurate and acceptable in treatment groups. Furthermore, we showed that synthesized biocompatible magneto-plasmonic nanohybrid (the AuNRs coated with BSA as a biocompatible NIR-absorbing agent and Fe_3_O_4_@CMD as an MRI contrast agent) could be a promising agent for image-guided NPTT. We believe that our developed model based on the synthesized nanohybrid should yield more accurate prediction of temperature distribution, which opens new avenues to clinical applications.

## Materials and methods

### Materials

Tetra chloroauric acid (HAuCl_4_ × 3H_2_O), ammonium hydroxide (NH_4_OH, 28%), Roswell Park Memorial Institute (RPMI) 1640 cell culture medium, 1-ethyl-3-[3-dimethylaminopropyl] carbodiimide (EDC), *N*-hydroxy succinimide (NHS), dialysis membranes with a molecular mass cut-off at 20 kDa, penicillin–streptomycin, CTAB, dextran, iron(II) chloride tetrahydrate (FeCl_2_·4H_2_O, 99%), iron(III) chloride hexahydrate (FeCl_3_·6H_2_O, 99%), NaOH, bromoacetic acid, ammonium hydroxide (5 M), BSA and trypsin–ethylene diamine tetraacetic acid (EDTA) were obtained from Sigma-Aldrich Company (USA). Fetal bovine serum (FBS) was purchased from Gibco^®^ (USA). All described materials were utilized for cell culture experiments and nanohybrids synthesis. Other chemicals and reagents were of analytical grade and commercially available.

### Cell culture, cytotoxicity assay and animal model

CT26 murine colorectal carcinoma cell line and male BALB/c mice were purchased from the Pasteur Institute of Iran. Cells were cultured in RPMI 1640 medium with 10% FBS, 100 µg/ml streptomycin, and 100 units/ml penicillin at 37 °C in an atmosphere with 5% CO_2_. Cells were harvested by trypsinizing cultures with trypsin–EDTA. The viability of CT26 cells treated with nanohybrids at varying gold concentrations over 4, 12, and 24 h presented in the Additional file 1. For animal model establishment, 200 µl of CT26 cell line suspension (2 × 10^6^ cells per 200 µl RPMI) were injected subcutaneously into the right flank of mice (5 − 7 weeks old, 17 − 20 g) to establish the tumor model. All the animal experiments were conducted in accordance with the approval obtained from ethics committee of Iran University of Medical Sciences (IUMS) (Ethics Code: IR.IUMS.FMD.REC.1399.544).

### Synthesis of AuNRs@BSA-Fe_3_O_4_@CMD

The preparation procedure of nanohybrid consisted of three steps: synthesis of AuNRs@BSA, synthesis of Fe_3_O_4_@CMD, and attaching the AuNRs@BSA to the Fe_3_O_4_@CMD.

### AuNRs@BSA

The seed-mediated synthesis of AuNRs was performed as described by El-Sayed et al., with some minor modifications [39]. Briefly, the CTAB-coated gold seeds (~ 4 nm) were prepared by the chemical reduction method. A solution of 0.25 ml gold salt (10 mM) was mixed with a 7.5 ml CTAB solution (100 mM) in a 25-ml round bottom balloon under stirring, then ice-cold NaBH_4_ solution (0.6 mL, 10 mM) was added. The solution color immediately turned yellow–brown, indicating the formation of AuNPs. Second, growth solution was prepared by adding CTAB (1 l, 100 mM), chloroauric acid (HAuCl_4_, 50 ml, 10 mM), silver nitrate (AgNO_3_, 9 ml, 10 mM), and sulfuric acid (20 ml, 0.5 mM) to 2000-mL round bottom flask. The equilibrium was achieved under stirring for 30 min at 30 °C. Afterward, ascorbic acid (8 ml, 100 mM) was added under a strong stirring. The solution was decolorized immediately, then the synthesized gold seeds (2.5 ml) were added under stirring for 2 min, then incubated at 30 °C for 6 h. Finally, to replace the CTAB coating with BSA [40], the CTAB-coated AuNRs were subjected to two centrifuge/washing cycles (12,000 rpm, 10 min) to remove the CTAB. Subsequently, they were stirred in a solution of BSA (100 ml, 0.2 g/ml) for 12 h at room temperature. After the incubation, the mixture was centrifuged twice as a final washing step.

### Fe_3_O_4_@CMD

First, for CMD synthesis [41], 1.0 g of dextran was dissolved in 3 ml of distilled water. Then, 3 ml of sodium hydroxide solution (NaOH, 8 M) was added, and the temperature was raised to 60–65 °C in a water bath. Afterward, 0.4 g of bromoacetic acid (C_2_H_3_BrO_2_) was added, and the reaction continued at this temperature for 2 h, and then the reaction solution was neutralized by adding acid. Next, dextran was precipitated by ethanol, washed three times, and finally dried in the oven at 60 °C. Second, 1.0 g of CMD was dissolved in 25 ml of water and passed through a 0.2-µm syringe filter to obtain Fe_3_O_4_@CMD. 0.75 g of FeCl_3_.6H_2_O salt and 0.4 g of FeCl_2_.4H_2_O salt was dissolved in 10 ml of water and filtered by a 0.2-µm syringe filter. Then, CMD and iron salts solutions were mixed, and the precipitation was dissolved by the lowest possible amount of hydrochloric acid (HCl, 2 M). The temperature of the solution was reduced to 10 °C, and 2.83 ml of 28% ammonia was added gradually dropwise. The resulting product was maintained at 78 °C for 1 h. Then, it was cooled down to room temperature and neutralized with acetic acid. Large particles were separated with centrifugation at 3500 rpm, and the suspension was then dialyzed for one day with a dialysis bag (cut-off 20 kDa).

### Attachment of Fe_3_O_4_@CMD to AuNRs@BSA

**Fig. 9:**
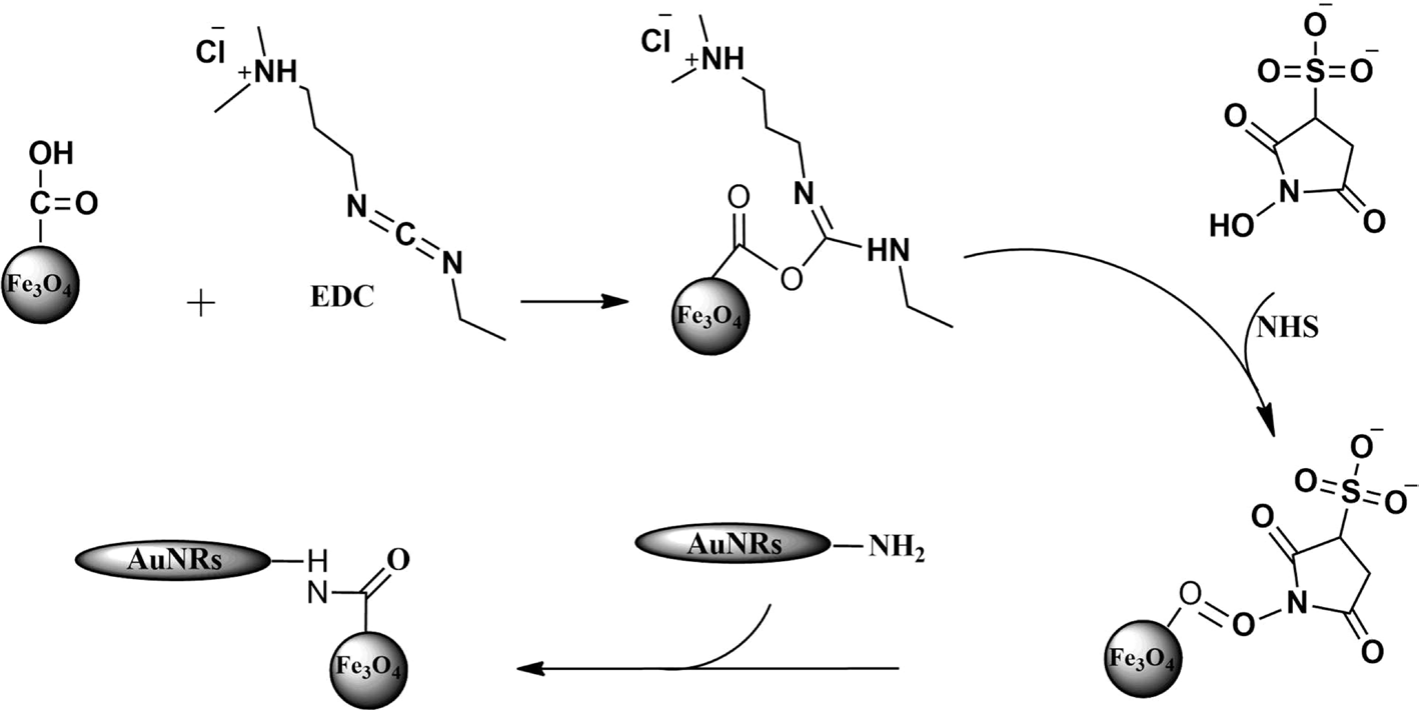
Schematic drawing of the possible bioconjugation reaction between Fe_3_O_4_@CMD and AuNRs@BSA, in the presence of EDC and NHS. Nucleophilic attack by NH_2_ group of BSA leads to the formation of an amide bond between COOH group of CMD and NH_2_ group of BSA. EDC and NHS catalyze the covalent bindings between amino acid and carboxyl groups, thus, crosslinking AuNRs@BSA with the Fe_3_O_4_@CMD and forming AuNRs@BSA-Fe_3_O_4_@CMD

Figure 9 shows the conjugation of AuNRs@BSA via their amino groups to the carboxyl groups of Fe_3_O_4_@CMD through the EDS/NHS amine coupling reaction [27]. A mixture of Fe_3_O_4_@CMD (4000 mg l^−1^, 1000 µl), EDC (0.5 mg), and NHS (0.3 mg) were prepared in an aqueous solution (pH 6–7) and stirred for 30 min at room temperature. Then, AuNRs@BSA was added and stirred for 2 h at room temperature. The resulting mixture was centrifuged for 10 min at 10,000 rpm three times, and then the volume reached 1 ml.

### Characterization techniques

The UV–visible absorption of samples was measured over the wavelength ranging from 450 to 1100 nm with a UV–visible spectrophotometer (BioMate 5, Thermo, USA). The zeta potential of the solutions was measured by using a ZETA-check (Analytik, UK). For FTIR analysis, the powdered samples were analyzed by FTIR spectrophotometer (AVATAR, Thermo, USA) in the range of 4000–400 cm^−1^. TEM characterization was performed using a TEM machine (CM30, Philips, Netherlands) operating at 250 kV, and images were used to obtain the size distribution of particles using ImageJ (1.53 k) software. XRD analysis was done using an X-ray diffractometer (PANalytical 'X'Pert Pro, Netherlands) equipped with a Cu Kα source (λ = 0.154060 nm). The 2θ angles were collected from 20° to 78°, step size of 0.04°.

### PCE of the nanohybrids and in vitro study of NPTT

The PCE of plasmonic metal nanoparticles is a crucial parameter in NPTT. The formula and outcomes of this evaluation are presented in the Additional file 1. Subsequently, as determined by MTT assay, we employed 20 μg/ml gold concentration of nanohybrids as a photothermal agent for in vitro cancer cell ablation under laser irradiation. Our findings showed that a majority of cells were destroyed under 808-nm NIR laser at 1 W/cm^2^ for 5 min. The methods and results for this assay are also included in the Additional file 1.

### In vivo MRI-guided NPTT

Fifteen mice were selected for NPTT. CT26 tumor-bearing mice were randomly divided into three groups (*n* = 5 per group) and underwent MR imaging. For IP injection, nanohybrids (250 µl; 7.5 mg Fe/kg, 3.75 mg Au/kg) were injected and imaged after 24 h. For IT injection, nanohybrids (25 µl; 500 μg/ml Fe, 250 μg/ml Au) were injected directly into the tumor, and the mice were then immediately imaged post-injection. Following imaging, the treatment groups, namely laser alone, IP injection + laser, and IT injection + laser were irradiated with the 808 nm NIR laser at a power density of 1.4 W/cm^2^ for 5 min at the tumor site. Tumor volume was measured using a digital caliper at the widest width and perpendicular length, and calculated using the formula: tumor volume = (tumor length) × (tumor width)^2^/2. All MRI images were obtained at the national brain-mapping lab, Tehran, Iran. MRI images were acquired using a Siemens MAGNETOM Prisma (Siemens, Germany) 3T scanner equipped with an animal coil. All anatomical T2-weighted image acquisitions were performed using the following parameters: TR/TE = 4400/110, slice thickness: 0.7 mm, matrix size: 256 × 256, and FOV: 20 × 10 cm.

### ICP-MS analysis

After MR imaging, to analyze the gold content weight of accumulated nanohybrids in the tumor, the IP injection group was killed after 24 h. Then, the tumor region was dissected and lysed with aqua regia overnight. The experiment was performed by ICP-MS analysis, ICP-MS (ELAN 6100 DRC, PerkinElmer, Canada).

### Image segmentation and generating the 3D model of mice

The acquired DICOM-formatted MRI scan images were imported into 3D Slicer software for image processing [42]. Manual segmentation and contouring were performed based on grayscale values to generate the geometry of 3D model according to the following structures defined by the project: the hypointense volume that shows the accumulation of nanohybrids in the tumor and tumor-bearing mice whole body. These masks were further processed to improve the quality of the segmentation for mesh generation and fixed overlaps and mesh errors. The meshes were optimized in Gmsh software for simulation by undergoing adaptive quality optimization cycles to ensure various mesh quality targets were met for importing into COMSOL [43]. The model consisted of 1.2 million tetrahedral elements. Finally, the finite element mesh was exported into COMSOL Multiphysics for model development and simulation.

### Theoretical model

The external heat source is a function of the absorption μ_an_ and scattering μ_sn_ coefficients of nanohybrids, Eqs. (1, 2). To this end, we derived these parameters from Mie-electrostatic approach, which can be described as [44]:

1 $${\mu }_{\mathrm{an}}=\frac{2\pi {f}_{\mathrm{V}}}{\lambda {V}_{\mathrm{np}}}\mathrm{imag}\left(\frac{{a}_{1}}{3}+\frac{{a}_{2}}{3}+\frac{{a}_{3}}{3}\right),$$

2 $${\mu }_{\mathrm{sn}}=\frac{16{\pi }^{3}{f}_{\mathrm{V}}}{18{\lambda }^{4}{V}_{\mathrm{np}}}\left({\left|{a}_{1}\right|}^{2}+{\left|{a}_{2}\right|}^{2}+{\left|{a}_{3}\right|}^{2}\right),$$

where *f*_V_ is the volume fraction of nanohybrids in the tumor-bearing mice, λ is the wavelength of the laser, *V*_np_ is a single AuNR volume, and polarization terms ${a}_{1}$, ${a}_{2}$ and ${a}_{3}$ are defined as:

3 $${a}_{i}=4\pi {D}^{2}l\left(\frac{\varepsilon -{\varepsilon }_{m}}{3{P}_{i}\left(\varepsilon -{\varepsilon }_{m}\right)+3{\varepsilon }_{m}}\right),$$

where ε is the dielectric function of the gold, which was taken from the famous report by Johnson and Christy [45], ε_m_ is surrounding medium dielectric constant, and *P*_i_ is geometry factor that calculated by the following equation:

4 $${P}_{1}=\frac{1-{\beta }^{2}}{{\beta }^{2}}\left(\frac{1}{2\beta }{\mathrm{Ln}}\left(\frac{1+\beta }{1-\beta }\right)-1\right), {P}_{2}={P}_{3}=\frac{1-{P}_{1}}{2} , \beta ={D}/{l},$$

where *D* and *l* are the diameter and length of the AuNRs, respectively.

**Fig. 10:**
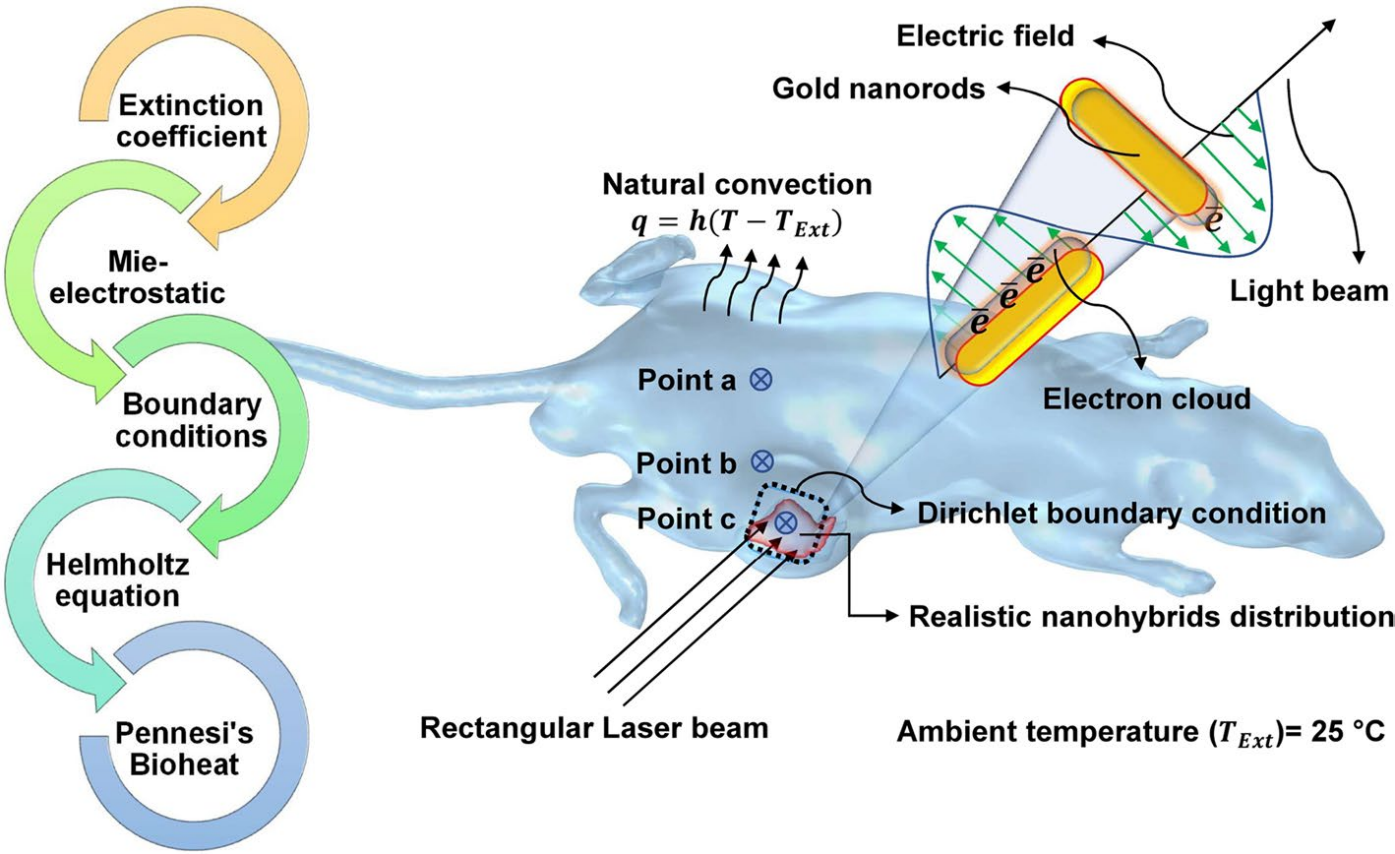
Outline of the modeling, and boundary conditions

The fluence rate of laser in tissue loaded by nanohybrids was calculated based on the transport theory of laser propagation, which performs the radiative transport equation to calculate light propagation vectors. To solve the radiative transport equation, we supposed that the radiance is isotropic because the derived partial differential is significantly easier to solve. Hence, the light diffusion is described by the following equations:

5 $$\nabla \left(-C\nabla u\right)+{\mathrm{au}}=f,$$

6 $$C =\frac{1}{3( {\mu }_{\mathrm{s}}^{^{\prime}} + {\mu }_{\mathrm{a}})},$$

7 $${\mu }_{\mathrm{a}}={\mu }_{\mathrm{at}}+{\mu }_{\mathrm{an}},$$

8 $$\left\{\begin{array}{c}{\mu }_{\mathrm{s}}={\mu }_{\mathrm{st}}+{\mu }_{\mathrm{sn}}\\ {\mu }_{\mathrm{s}}^{^{\prime}}={\mu }_{\mathrm{s}}\left(1-g\right)\end{array}\right.,$$

where μ_a_ is the total absorption coefficient (cm^−1^), ${\mu }_{\mathrm{s}}^{^{\prime}}$, is the total reduced scattering coefficient (cm^−1^), μ_at_ is absorption coefficient (cm^−1^) of tumor, μ_st_ is scattering coefficient (cm^−1^) of tumor, and *g* is the tissue anisotropy factor. Simulations were performed using the values obtained from the relevant literature listed in Table 1. Furthermore, Fig. 10 depicts the boundary conditions and outline of the modeling process.

**Table 1:** List of parameters with numerical values and units used for simulation

Parameters	Values	Units	Source
*ρ* (density of tissue)	1052	kg m^−3^	[20]
*C* (specific heat of tissue)	3800	J kg^−1^ k^−1^	[20]
*K* (thermal conductivity)	0.545	W m^−1^ k^−1^	[20]
ρ_b_ (blood density)	1052	kg m^−3^	[20]
*C*_b_ (specific heat of blood)	3800	H	[20]
ω_b_ (blood perfusion)	0.01	1/s	[20]
*h* (convective coefficient)	10	W m^−2^ k^−1^	[20]
μ_at_ (absorption coefficient of tissue)	2	cm^−1^	[46]
μ_st_ (scattering coefficient of tissue)	61	cm^−1^	[46]
μ_an_ (absorption coefficient of nanohybrids)	54	cm^−1^	This study
μ_sn_ (scattering coefficient of nanohybrids)	0.0007	cm^−1^	This study
Ea (activation energy)	7.39E+39	J/mol	[21]
*A* (frequency factor)	2.50E+05	s^−1^	[21]
*f*_Vi_ (IT injection volume fraction)	1E−07	–	This study
*f*_Vp_ (IP injection volume fraction)	5E−09	–	This study
*g* (tissue anisotropy factor)	0.67	cm^−1^	[20]

The theoretical model, describing the response to photothermal therapy mediated by nanohybrids, is based on the Pennes' bio-heat equation. The main mechanism of heat transfer into the tissue is mathematically expressed by [20]:

9 $$\rho C\frac{\partial T(r,t)}{\partial t}=\nabla \left(k\nabla \left(r,t\right)\right)+{Q}_{\mathrm{b}}+{Q}_{\mathrm{m}}+{Q}_{\mathrm{ext}},$$

where ρ is tissue density (kg m^−3^), *C* is tissue specific heat (J kg^−1^ C^−1^), and *k* is conductivity (W m^−1^ C^−1^). The dependent variable *T*(*r*,*t*) is the tissue temperature expressed as a function of spatial coordinate (*x*, *y*, *z*) and time (*t*). For simplicity, tissue is assumed to be homogeneous and isotropic, and the geometry was modeled considering cylindrical symmetry. *Q*_b_ the heat contribution due to blood perfusion per volume unit expressed by the following:

10 $${Q}_{\mathrm{b}}={\rho }_{\mathrm{b}}{C}_{\mathrm{b}}{\omega }_{\mathrm{b}}\left(T\left(r,t\right)-T\left(b\right)\right),$$

where ρ_b_ is blood density (kg m^−3^), *C*_b_ is the blood specific heat (J kg^−1^ k^−1^), ω_b_ is the blood perfusion rate (s^−1^) and *T*(*b*) is the blood temperature outside the treatment site. *Q*_m_ is the metabolic heat generation due to the oxidative process of lipids, proteins, and carbohydrates. *Q*_ext_ is absorbed laser energy (W m^−3^) in the target, and calculated by the following equation [47]:

11 $${Q}_{\mathrm{ext}}\left(r,t\right)={\mu }_{\mathrm{a}}u\left(r,t\right).$$

To consider the heat loss at the surface boundary of the mouse body due to the convection heat flux mechanism, a Neumann boundary condition was applied, which was expressed as follows:

12 $$k\frac{\partial T(0,t)}{\partial n}=h\left({T}_{\infty }-{T}_{0}\right),$$

where *k* is the tissue conductivity (W m^−1^ k^−1^), ∂*T*/∂*n* is the temperature gradient at the skin surface (K m^−1^), h is the heat transfer coefficient, *T*_∞_ is the temperature at the surface boundary, and *T*_0_ is ambient temperature of the environment (25 °C). Finally, we applied the Dirichlet boundary condition on the tumor surface as follows:

13 $$-{\varvec{n}} . \left(-C\nabla u\right)+\mathrm{au}=0,$$

where *n* is a unit normal vector. The prescribed value of light fluence rate was set to 1.4 (W/m^2^), and zero flux was applied for out of the target.

### Thermal damage model

The Arrhenius equation (Eq. 14) was used to describe the irreversible thermal damage. The first order of the chemical rate equation, which represents the tissue between two different states, is calculated by [48]:

14 $$\Omega \left(\tau \right)={\int }_{0}^{\tau }A\mathrm{exp}\left(-\frac{{E}_{a}}{\mathrm{RT}(t)}\right)\mathrm{d}t,$$

where Ω(τ) is the thermal damage rate of tissue, *A* is frequency factor (s^−1^), *E*_a_ is the activation energy (kJ mol^−1^), and *R* is the gas constant (8.314 J K^−1^ mol^−1^). T and t stand for the absolute temperature of tissue and duration of heat exposure, respectively. The value of Ω(τ) was calculated with COMSOL at each time step of a thermal profile.

### Histologic assessments and staining

After MR imaging, the mice were killed immediately. The tumor and selected organs including liver, lung, spleen, and kidney were taken out and immersed in 10% buffered formalin solution for 72 h. Then, the paraffin-embedded sections were made and stained with Prussian blue staining method to verify the presence of iron in the tissues [49]. Prepared samples were examined under a light microscope (magnification 40×).

### Statistical analysis

Experiments were performed in triplicate, and all values were presented as the mean ± SD. Differences between indicated groups were analyzed using a two-tailed Student's *t*-test available in RStudio. A *p*-value < 0.05 was considered statistically significant.

## Supplementary Information

**Additional file 1: Figure S1 a** Plot of the temperature vs. time for the nanohybrids (500 μg/ml Au, A_808_ = 3.2) during laser irradiation (808 nm, 1.4 W/cm^2^) and cooling (laser off) stages. (b) Plot of the cooling time vs. -Lnθ. **Figure S2** In vitro cell experiments. **a** Viability of CT26 cells treated with nanohybrids at varying gold concentrations over 4, 12, and 24 hours. **b** Viabilities of CT26 cells after NPTT at different laser power densities. Error bars were based on the standard deviations of three parallel samples.

## Data Availability

All data generated or analyzed during this study are included in this published article.
