# Supplementary material for: 3D modeling of in vivo MRI-guided nano-photothermal therapy mediated by magneto-plasmonic nanohybrids
Source: Biomed Eng Online. 2023 Aug 1;22:77. doi: 10.1186/s12938-023-01131-w (PMC10394893; doi:10.1186/s12938-023-01131-w)
Supplement: Supplementary file 1 — Additional file 1: Figure S1 a Plot of the temperature vs. time for the nanohybrids (500 μg/ml Au, A808 = 3.2) during laser irradiation (808 nm, 1.4 W/cm2) and cooling (laser off) stages. (b) Plot of the cooling time vs. -Lnθ. Figure S2 In vitro cell experiments. a Viability of CT26 cells treated with nanohybrids at varying gold concentrations over 4, 12, and 24 hours. b Viabilities of CT26 cells after NPTT at different laser power densities. Error bars were based on the standard deviations of three parallel samples. [file 12938_2023_1131_MOESM1_ESM.docx]

**Additional file 1**

**3D modeling of *in vivo* MRI guided nano-photothermal therapy mediated by magneto-plasmonic nanohybrids**

Zahed Tavangari^1,2^, Mohammadreza Asadi^1^, Rasoul Irajirad^1^, Abolfazl Sarikhani^1,2^,
Zahra Alamzadeh^1^, Habib Ghaznavi^3,*^, Samideh Khoei^1,2,*^

*^1^Finetech in Medicine Research Center, Iran University of Medical Sciences, Tehran, Iran*

*^2^Medical Physics Department, Iran University of Medical Sciences, Tehran, Iran*

*^3^Pharmacology Research Center, Zahedan University of Medical Sciences, Zahedan, Iran*

(*) ***Corresponding authors:***

H. Ghaznavi: [dr.ghaznavi@zaums.ac.ir](mailto:dr.ghaznavi@zaums.ac.ir)

*Pharmacology Research Center, Zahedan University of Medical Sciences, Zahedan, Iran*

S. Khoei: [khoei.s@iums.ac.ir](mailto:khoei.s@iums.ac.ir)

*Finetech in Medicine Research Center, Iran University of Medical Sciences, Tehran, Iran*

*Telephone:+98 21 8862 2647*

**Calculation of the photothermal conversion efficiency**

A home-built setup was employed to irradiate the nanohybrids with laser and measure the photothermal conversion efficiency. The setup consisted of a cuvette with a 1 cm path length, containing 1 ml of the nanohybrid solution (500 μg/ml Au), an infrared (IR) thermal imaging camera (Testo 875−1i, Germany), and an 808 nm continuous semiconductor diode laser. The photothermal conversion efficiency of the magneto-plasmonic nanohybrids was determined using the formula [1]:

$\eta=\frac{hs(T_{max}-T_{surr})-Q_{dis}}{I(1-{10}^{-A_{808nm}})}$ (1)

Here, h, s, T_max_, T_sur_, I, A_808_, and Q_dis_ represent the heat transfer coefficient, the surface area of the container, the maximum equilibrium temperature, the surrounding temperature, the laser power, the absorbance nanohybrids at 808 nm, and Q_dis_ is heat dissipation due to light absorbance of the solvent, which can be calculated according to Equation 2:

$Q_{dis}=\frac{mC\Delta T}{t}$ (2)

The variables m, C, ΔT, and t represent, respectively, the mass of water in grams (g), the specific heat capacity of water (J.g^-1^.K^-1^), the temperature rise (K), and the duration of laser irradiation in seconds (s). hs can be calculated according to Equation 3:

$hs=\frac{\sum m_{i}c_{p,i}}{\tau_{s}}$ (3)

The symbols τ_s_ and i are used to denote the time constant of the sample and the individual components of the system, which include the nanohybrids suspension and cuvette. τ_s_ can be calculated according to Equation 4:

$\left\{ \begin{aligned} \tau_{s}=\frac{t}{-ln \left( \theta\right)} \\ \theta=\frac{\left( T-T_{surr} \right)}{\left( T_{max}-T_{surr} \right)} \end{aligned} \right.$ (4)

In this study, the temperature variation of the nanohybrid solution was monitored during the heating stage using laser irradiation, followed by subsequent cooling without the use of laser irradiation (Fig. S1a). After 11 minutes of irradiation, the maximum temperature of the nanohybrid solution increased by 28.6 °C, which can be attributed to the thermal equilibrium between the heat input and output. The time constant for heat transfer, τ_s_, was determined by linear fitting of the cooling time t versus the natural logarithm of the temperature decrease (-lnθ) and was found to be 5.43 minutes (Fig. S1b). According to the given information, Q_dis_ was calculated to be 0.53 mW using Equation (2). The photothermal conversion efficiency (η) of the nanohybrids was estimated to be approximately 30%. In order to assess the photothermal stability of the nanohybrid, a series of reversible photothermal heating/cooling experiments were conducted (Fig. S1a). The results demonstrated that repeated laser irradiation did not cause any significant changes in the photothermal performance of the synthesized nanohybrid.


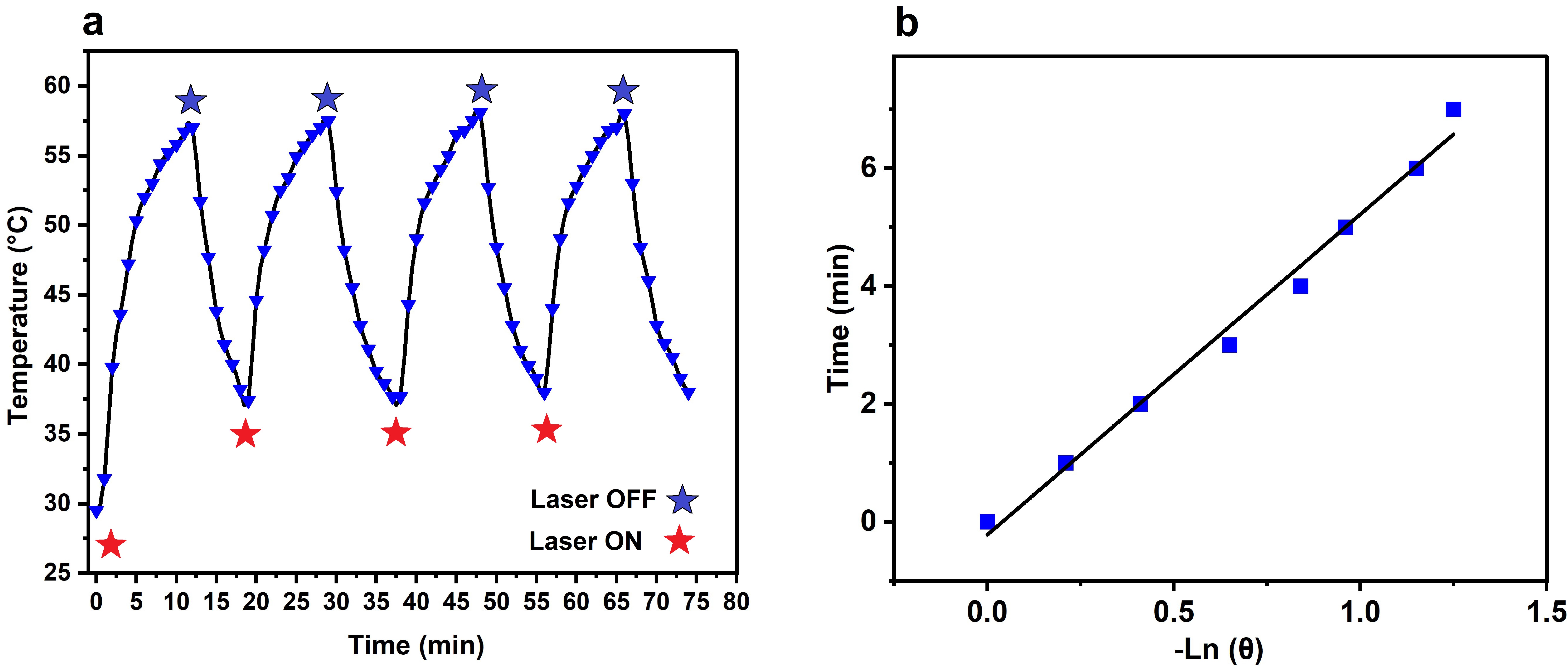


**Fig. S1** **a** Plot of the temperature vs. time for the nanohybrids (500 μg/ml Au, A_808_ = 3.2) during laser irradiation (808 nm, 1.4 W/cm^2^) and cooling (laser off) stages. (b) Plot of the cooling time vs. -Lnθ.

***In vitro* study of NPTT**


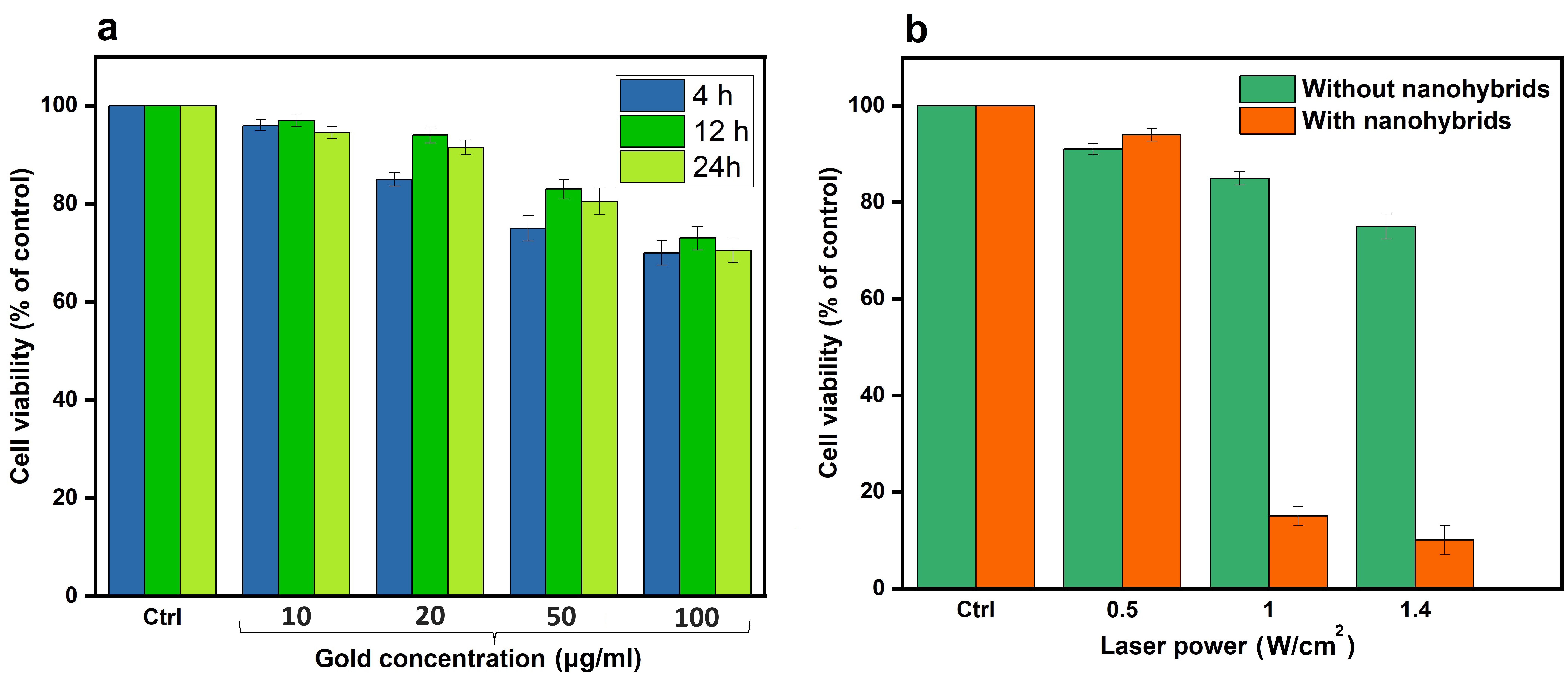
 We assessed the cytotoxicity of the nanohybrids using the methyl thiazolyl tetrazolium (MTT) assay. Briefly, we seeded a suspension of CT26 cells at a concentration of 5 × 10^4^ cells/ml in complete RPMI medium into a 96-well plate and allowed them to adhere for 24 hours. We then replaced the medium with a fresh one containing nanohybrids with varying gold concentrations. After incubation for 4, 12, and 24 hours at 37°C, we added 50 μl of MTT solution (1.0 mg/ml in PBS) to each well. Following an additional 4 hours of incubation, we removed the medium and added 100 μl of dimethyl sulfoxide (DMSO) to each well to dissolve the formazan crystals. We measured the absorbance of the formazan crystals at 570 nm using a Bio-Rad ELISA reader. Fig. S2a shows the viability of CT26 cells treated with nanohybrids at varying gold concentrations over 4, 12, and 24 hours. Based on MTT assay results, CT26 cancer cells were incubated with nanohybrid at the concentration of 20 μg/ml for 12h and then irradiated by the 808 nm laser at different laser power densities for 5 min. An MTT assay was also performed to quantitatively measure the cell viabilities after NPTT under different laser powers (Fig. S2b).

**Fig. S2** *In vitro* cell experiments. **a** Viability of CT26 cells treated with nanohybrids at varying gold concentrations over 4, 12, and 24 hours. **b** Viabilities of CT26 cells after NPTT at different laser power densities. Error bars were based on the standard deviations of three parallel samples.

**References**

1. Tian Q, Jiang F, Zou R, Liu Q, Chen Z, Zhu M, Yang S, Wang J, Wang J, Hu J: **Hydrophilic Cu9S5 nanocrystals: a photothermal agent with a 25.7% heat conversion efficiency for photothermal ablation of cancer cells in vivo.** *ACS nano* 2011, **5**(12):9761-9771.
